# Single-cell RNA-seq data analysis reveals functionally relevant biomarkers of early brain development and their regulatory footprints in human embryonic stem cells (hESCs)

**DOI:** 10.1093/bib/bbae230

**Published:** 2024-05-12

**Authors:** Md Alamin, Most Humaira Sultana, Isaac Adeyemi Babarinde, A K M Azad, Mohammad Ali Moni, Haiming Xu

**Affiliations:** Shenzhen Key Laboratory of Gene Regulation and Systems Biology, Department of Biology, School of Life Sciences, Southern University of Science and Technology, Shenzhen 518055, Guangdong, China; Institute of Bioinformatics, Zhejiang University, Hangzhou 310058, China; Shenzhen Key Laboratory of Gene Regulation and Systems Biology, Department of Biology, School of Life Sciences, Southern University of Science and Technology, Shenzhen 518055, Guangdong, China; Department of Mathematics and Statistics, College of Science, Imam Muhammad Ibn Saud Islamic University, Riyadh 11432, Saudi Arabia; Artificial Intelligence and Cyber Futures Institute, Charles Sturt University, Bathurst, NSW 2795, Australia; Institute of Bioinformatics, Zhejiang University, Hangzhou 310058, China

**Keywords:** single-cell RNA sequencing, differentially expressed genes, neuronal development, human embryonic stem cells, bioinformatics

## Abstract

The complicated process of neuronal development is initiated early in life, with the genetic mechanisms governing this process yet to be fully elucidated. Single-cell RNA sequencing (scRNA-seq) is a potent instrument for pinpointing biomarkers that exhibit differential expression across various cell types and developmental stages. By employing scRNA-seq on human embryonic stem cells, we aim to identify differentially expressed genes (DEGs) crucial for early-stage neuronal development. Our focus extends beyond simply identifying DEGs. We strive to investigate the functional roles of these genes through enrichment analysis and construct gene regulatory networks to understand their interactions. Ultimately, this comprehensive approach aspires to illuminate the molecular mechanisms and transcriptional dynamics governing early human brain development. By uncovering potential links between these DEGs and intelligence, mental disorders, and neurodevelopmental disorders, we hope to shed light on human neurological health and disease. In this study, we have used scRNA-seq to identify DEGs involved in early-stage neuronal development in hESCs. The scRNA-seq data, collected on days 26 (D26) and 54 (D54), of the *in vitro* differentiation of hESCs to neurons were analyzed. Our analysis identified 539 DEGs between D26 and D54. Functional enrichment of those DEG biomarkers indicated that the up-regulated DEGs participated in neurogenesis, while the down-regulated DEGs were linked to synapse regulation. The Reactome pathway analysis revealed that down-regulated DEGs were involved in the interactions between proteins located in synapse pathways. We also discovered interactions between DEGs and miRNA, transcriptional factors (TFs) and DEGs, and between TF and miRNA. Our study identified 20 significant transcription factors, shedding light on early brain development genetics. The identified DEGs and gene regulatory networks are valuable resources for future research into human brain development and neurodevelopmental disorders.

## INTRODUCTION

Genome-wide association studies have identified numerous single nucleotide polymorphisms linked to complex traits, including diseases, in humans [1]. Single-cell genomics and transcriptomics are the most robust and widely used tools to investigate single-cell biology at a genome-wide level [2]. Single-cell RNA-seq (scRNA-seq) allows the instantaneous and impartial estimation of gene expressions in cellular structures and particular cell categories [3, 4] and is exceptionally well-placed to explore uncommon cell categories [5, 6]. The brain and other organs in the central nervous system are composed of various cells, but many stay to be discovered due to the brain's vast complexity. The complexity in the brain and other tissues is established during embryogenesis. Human embryogenesis is related to the fascinating and diligent changes in cellular conditions, and the investigation of the molecular consequence of the genetic modifications in earlier human brain development has been greatly enhanced by scRNA-seq technology [7].

Human embryonic stem cells (hESCs) are pluripotent stem cells obtained from human embryos, which can differentiate into various cell types, including neurons. Researchers identified transcription factors (TFs) associated with cell states and state alterations in cell clusters and studied the lineage trees using hESCs [8, 9]. Studies involving hESCs have explored their role in brain development. When hESCs are introduced into the brain ventricles of embryonic mice, they can undergo differentiation to form functional neural lineages, contributing to brain development [10]. hESCs have been studied as a model for neural development and neurological diseases, including early-onset neurological disorders [11]. Although there is a limited number of clinical trials involving the use of embryonic or fetal stem cells for neurological disorder treatments, preclinical investigations using disease models have generated substantial evidence affirming the viability and effectiveness of stem cell therapies [12, 13]. Scientists have discovered that induced pluripotent stem cells, which are generated from a patient's own cells, can be differentiated into various neuron types, such as dopaminergic neurons. This breakthrough has allowed for the study and treatment of genetic diseases, particularly neurological disorders [14]. Clinical trials of the application of stem cells as a therapeutic approach to tackle various neurological disorders, encompassing conditions such as injuries affecting the brain, spinal cord and peripheral nerves, are now being performed [15]. Also, stem cell-derived motor neurons have aided in the discovery of amyotrophic lateral sclerosis drugs [16]. These investigations underscore the potential of human embryonic stem cells for researching and addressing neurological disorders.

Studying early-stage neuron development during human embryonic brain expansion is vital for comprehending human brain development. Numerous studies have employed scRNA-seq to examine variations in gene expression within both hESCs and their differentiated derivatives. A scRNA-seq study analysed a high-efficiency hESC-endothelial cell induction system and identified differentially expressed genes (DEGs) involved in endothelial cell differentiation [17]. A recent study used scRNA-seq to examine the developmental path of cardiomyocytes derived from human pluripotent stem cells in engineered tissues, uncovering DEGs related to cardiac differentiation [18]. Another study used scRNA-seq to explore the transcriptional heterogeneity and expression alterations in limbal stem cells originating from hESCs [19]. Transcriptome analysis of hESC-derived lineage-specific progenitors by scRNA-seq identified novel regulators of hESC differentiation to definitive endoderm [20]. Another study found that different hESC lines exhibit distinct gene expression patterns, with these DEGs significantly enriched in developmental pathways [21]. A recent scRNA-seq study mapped the early differentiation stages of hESCs and identified the dynamic expression patterns and potential regulatory roles of long non-coding RNAs [22]. Another new study presents a comprehensive scRNA-seq analysis of hESC-derived retinal organoids across five developmental time points, revealing nine distinct cell populations and novel insights into photoreceptor genesis and cell–cell interactions within the developing human retina [23]. These studies exemplify scRNA-seq’s capacity for discerning crucial DEGs in hESC differentiation, elucidating the underlying molecular mechanisms of these processes.

**Figure 1:**
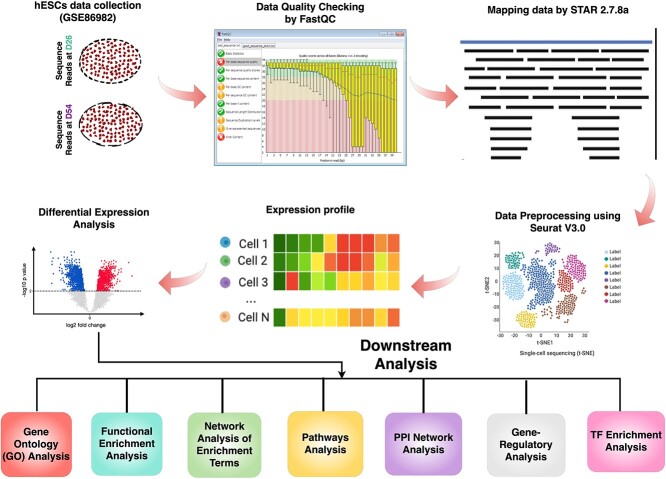
A schematic workflow for analyzing DEGs using scRNA-seq data in this study. The quality assessment of the data was first performed using FastQC. Next, the data was mapped to the human reference genome (hg38) using STAR. After mapping, the data was preprocessed using Seurat v3.0 to remove technical artifacts, normalize expression values, and identify highly variable genes. DEGs were performed to identify genes that were differentially expressed between the two-time points, D26 and D54. Finally, different downstream analyses were performed to gain a more reasonable interpretation of the biological functions of the genes that are expressed differently.

ScRNA-seq offers an unprecedented window into the dynamic world of hESCs as they differentiate into nascent neurons. We can illuminate the molecular programs producing early human brain development by pinpointing DEGs during this critical phase. Delving deeper, we can decipher the functional roles of these DEGs by exploring their enrichment in specific biological pathways and uncovering the intricate gene regulatory networks in which they participate. This knowledge may provide clues linking these genes to the foundation of human intelligence, shedding light on the origins of mental disorders and neurodevelopmental conditions. Ultimately, this investigation aspires to provide a comprehensive understanding of the molecular mechanisms and transcriptional dynamics governing the genesis of the human brain. However, there is still a significant gap in understanding the DEGs that lead to the early stages of hESC differentiation during brain development. This study addresses this gap by employing bioinformatics tools to pinpoint DEGs at two critical time points, day 26 (D26) and day 54 (D54), of neuronal differentiation from hESCs using scRNA-seq data. This study aims to first identify DEGs during early brain developmental stages, with the goal of revealing their functional enrichment of important and relevant pathways or functions, such as neurogenesis and synapse regulation or synapse-related interactions. Notably, these DEGs are investigated to highlight the enrichments in terms of intelligence, mental disorders, and neurodevelopmental disorders, providing valuable insights into potential connections between early brain development and cognitive outcomes. Furthermore, we explored the interplay between DEGs, microRNAs (miRNAs), and TFs to reveal their regulatory footprints on early neuronal development. Co-expression analysis is conducted to further illuminate the activity of TFs within the cerebellum region of the brain, adding a layer of specificity to the understanding of early neural development. This research is encapsulated in the data processing pipeline depicted in Figure 1. The findings presented here commend the prowess of scRNA-seq and beckon toward a deeper comprehension of the enigmatic world of early human brain development.

There are several advantages of this study. For example, we have used scRNA-seq to identify DEGs involved in early-stage neuronal development in hESCs, which is a powerful and unbiased method to capture the transcriptional heterogeneity and dynamics of single cells. We also employed three different statistical methods (MAST, Limma and DESeq2) to identify DEGs, which can reduce technical limitations and increase the accuracy of the results. Moreover, we have performed various downstream analyses to reveal the functional enrichment, gene regulatory networks, protein–protein interactions and transcription factor enrichment of the DEGs, which can provide valuable insights into the molecular mechanisms and biological implications of early brain development. However, our study has several limitations. For instance, we have only used two-time points (D26 and D54) to study early brain development, which may miss some important transitions and intermediate states of neuronal differentiation. Also, we did not perform any experimental validation or functional assays to confirm the roles of the DEGs, miRNAs and TFs in early brain development, which may limit the reliability and applicability of the findings. Furthermore, this study did not compare the results with other datasets or models of early brain development, which may overlook some common or specific features and potential sources of variation.

## METHODS AND MATERIALS

### scRNA-seq data collection and preprocessing

Openly accessible scRNA data of D26 (340 cells) and D54 (422 cells) profiled by SmatSeq2 were retrieved from the NCBI Gene Expression Omnibus (GSE86982). The SRR identities of all samples from the two-time points are available in Table S1, see Supplementary Data are available online at http://bib.oxfordjournals.org/. The quality of the downloaded data was assessed using FastQC and the read alignment to the human genome reference (hg38) was done by STAR 2.7.8a. Cells with fewer than $1\times{10}^6$ uniquely mapped reads and lower than 60% alignment rate were discarded from the analysis. Furthermore, cells with fewer than 3500 genes were removed. The expression levels of the filtered samples were normalized to counts per million.

### DEGs identification

We used a single-cell toolkit to pinpoint the DEG between D26 and D54 time points. The expression matrix was imported and converted into a Seurat object using the function ‘CreateSeuratObject’ from the Seurat package (Seurat v3.0). Then, quality control, normalization, feature selection, and clustering of marker genes of cells from scRNA-seq data were performed. We filtered out the cells with unique feature counts exceeding 10 000 or falling below 200 (Figure S1, see Supplementary Data are available online at http://bib.oxfordjournals.org/.). We also removed cells with mitochondrial and ribosomal counts greater than 20%. The ‘LogNormalize’ global-scaling normalization method was used to standardise individual cells’ feature expression measurements. Then, selection.method = ‘vst’ was used to find feature variables. Each gene's expression level was normalized by considering the total number of unique molecular identifiers (UMI) within each cell, followed by applying a natural logarithm to the UMI counts. Finally, we used three methods, MAST [24], limma [25], and DESeq2 [26], to remove the technical limitation of a single method to identify DEGs between D26 and D54. The thresholds for the final DEG set were FDR-corrected *P*-value <0.05 and absolute log2 fold change value >1.5.

### Gene annotation and functional enrichment analysis

Gene enrichment analysis of the DEGs was conducted using Metascape [27] with the default setting and clusterProfiler 4.0 [28]. Also, the protein–protein integration (PPI) network, DisGeNET, Cell Type Signatures, Transcription Factor Targets and transcriptional regulatory interaction network analysis were conducted using Metascape [27].

### P‌PI and gene regulatory network analysis

We relied on the STRING [29] protein interactome database to facilitate the PPI network analysis. We focused on identifying specific protein subnetworks enriched with DEGs. Following this, we created a PPI network using the DEGs we identified in our study.

Network Analyst [30] executed the DEG–miRNA, TF-DEGs and TF-miRNAs interaction network analyses. For Network Analyst, we utilized data from the miRTarBase [31] and TarBase [32] for DEG–miRNA analysis, ENCODE [33] for TF-DEGs and RegNetwork [34] for TF-miRNAs analysis. This integrated approach allows us to construct comprehensive and biologically relevant DEG–miRNA, TF-DEGs and TF-miRNAs interaction networks, facilitating a deeper understanding of regulatory mechanisms in our study.

### Transcription factor enrichment analysis

The ChEA3 tool [35] was utilized for deciphering the TFs responsible for observed gene expression alterations. The identified DEGs were supplied as the input for the transcription factor enrichment analysis in ChEA3. Subsequently, ChEA3 compared the discrete query gene sets with extensive TF target gene sets sourced from diverse 'omics' datasets, utilizing Fisher’s Exact Test with a reference size of 20 000 to identify TFs most closely linked to the input gene set. The output included enrichment results in tabular forms for each library and integration method, along with dynamically generated TF–TF co-regulatory networks based on top results.

## RESULTS

### Data description

We have acquired the transcriptomes of 762 single cells, which were profiled using the SmartSeq2 method. These cells originated from hESCs, and detailed information regarding the data generation and protocol can be found in previous studies [8, 9]. In the course of neural differentiation at various time intervals, targeted progenitor and neuron cells were isolated. These cells obtained through *in vitro* differentiation of hESCs cell lines containing SOX2cit/+ and DCXcit/Y genes were subjected to single-cell transcriptomic sequencing [9]. In this study, data collected on D26 and D54 were used to study the complexity of brain development. The average unique mapping rates were 82% and 73% for D26 and D54 cells, respectively. The average reads for D26 and D54 were 1 690 245 and 1 273 009, respectively. The average unique mappings reads for D26 were 1 391 903 and 967 180 for D54. The average multimapped read count for D26 was 79 182 and for D54 was 61 822.

### Identification of DEGs between D26 and D54

**Figure 2:**
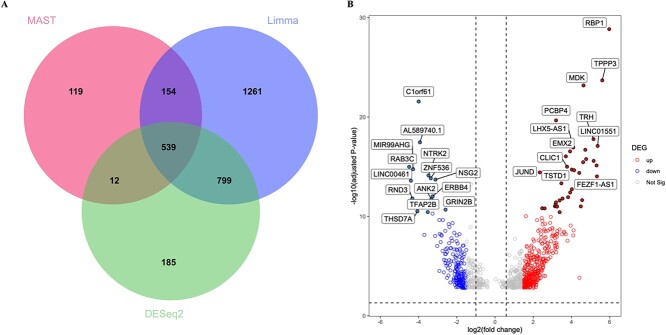
(A) Venn diagram using DEGs identified by MAST, limma and DESeq2. The common 539 DEGs were finally used in this study and (B) Volcano plot of the DEGs. Up-regulated, down-regulated and non-significant DEGs are indicated by the legend on the right side of the figure.

**Figure 3:**
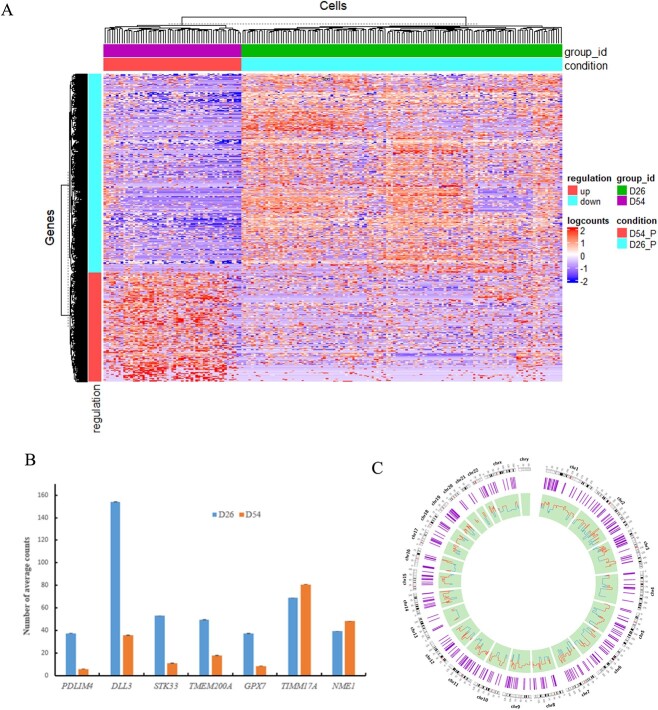
Comprehensive analysis of the identified DEGs. (A) Heatmap illustrating the expression patterns of DEGs, (B) Gene expression of selected DEGs at two-time points, and (C) Circos plot depicting the distribution and expression (log2 FC) values of DEGs across the 23 human chromosomes. The purple circle indicates DEGs distribution, and the light green represents their expression levels.

Detection of organized transcriptional changes is crucial in investigating scRNA-seq data [36]. To assess the transcriptional changes between D26 and D54, we used three methods, MAST, limma and DESeq2, to remove the technical limitation of a single method to identify DEGs and using the criteria of |log_2 FC| greater than 1.5 and FDR-corrected *P*-value less than 0.05. DEG analyses using MAST, limma and DESeq2 identified 824, 2753 and 1535 DEGs, respectively. We used the common 539 DEGs identified by all three methods for further analysis in our study (Figure 2A and Table S2, see Supplementary Data are available online at http://bib.oxfordjournals.org/). Among the 539 DEGs, 348 were down-regulated and 191 were up-regulated, as depicted in the volcano plot (Figure 2B and Table S2, see Supplementary Data are available online at http://bib.oxfordjournals.org/). Hierarchical clustering was conducted to visualize the similarities and differences in gene expression patterns between the two-time points (Figure 3A). To further validate the expression changes of DEGs, we randomly selected several DEGs. For example, *PDLIM4*, *DLL3*, *STK33*, *TMEM200A* and *GPX7* exhibiting significantly higher average gene expression levels at D26 than D54 (Figure 3B). Conversely, *TIMM17A* and *NME1* at D54 displayed significantly higher average gene expression levels compared with D26 (Figure 3B). These findings suggest that these genes have distinct roles at different time points during neuron development. Additionally, we observed that the distribution of up- and down-regulated DEGs at D26 and D54 spanned across all 22 chromosomes of the human genome, as illustrated in the Circos plot (Figure 3C). Our findings imply extensive transcriptional changes across diverse genomic regions. These insights into the differences between D26 and D54 shed light on key DEGs, particularly those pivotal in early neuron development, shaping brain development and potentially influencing neuron differentiation throughout the entire process.

### Revealing the timely evolution of gene expression profiling through single-cell analysis at D26 and D54

**Figure 4:**
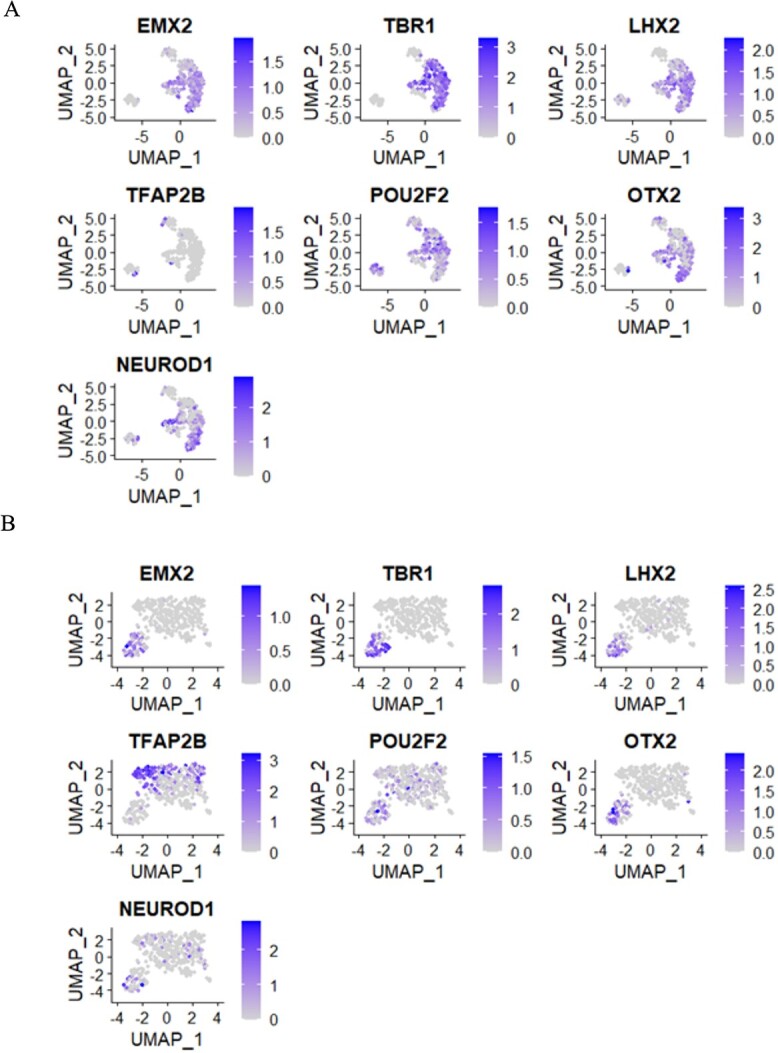
UMAP plot of the selected DEGs. (A-B) UMAP visualization of some selected genes at D26 and D54, respectively. These crucial genes act as master regulators, guiding neurogenesis, neuronal differentiation, and tissue development in the complex orchestration of brain formation.

In our study, we thoroughly analyzed gene expression profiles, including *NEUROD1*, *EMX2*, *TBR1*, *LHX2*, *POU2F2*, and *OTX2*, at D26 and D54, using single-cell analysis (Figure 4). These genes play a crucial role in regulating processes like neurogenesis, neuronal differentiation, and tissue development. For example, *NEUROD1* encodes the transcription factor protein NEUROD1, which plays a pivotal role in controlling gene expression and contributes to nervous system development [37]. *EMX2*, a homeobox gene, contributes to the development of the dorsal telencephalon and cortical development [38].

*TBR1*, known as T-Box Brain Transcription Factor 1, is classified as a protein-coding gene. It has been linked to medical conditions like intellectual developmental disorder, autism, and speech delay (www.genecards.org). *POU2F2* encodes the transcription factor protein Octamer-Binding Protein 2, which governs gene expression and is instrumental in the development of B cells, T cells, and neurons (www.genecards.org). *OTX2*, a homeobox gene, plays a vital part in the development of the forebrain and midbrain [38]. The differences in DEG expression at D26 and D54 provide key insights into the timing of gene activity, laying the foundation for a more profound comprehension of the mechanisms behind the developmental transitions we studied. Heatmap results showed a clear difference in gene expression levels between the two-time points using the different neuron development-related DEGs (Figure S2, see Supplementary Data are available online at http://bib.oxfordjournals.org/). These genes play a role in regulating processes like neurogenesis, cell differentiation, and tissue development.

### Functional enrichment analysis reveals transcriptional changes in neuron development

**Figure 5:**
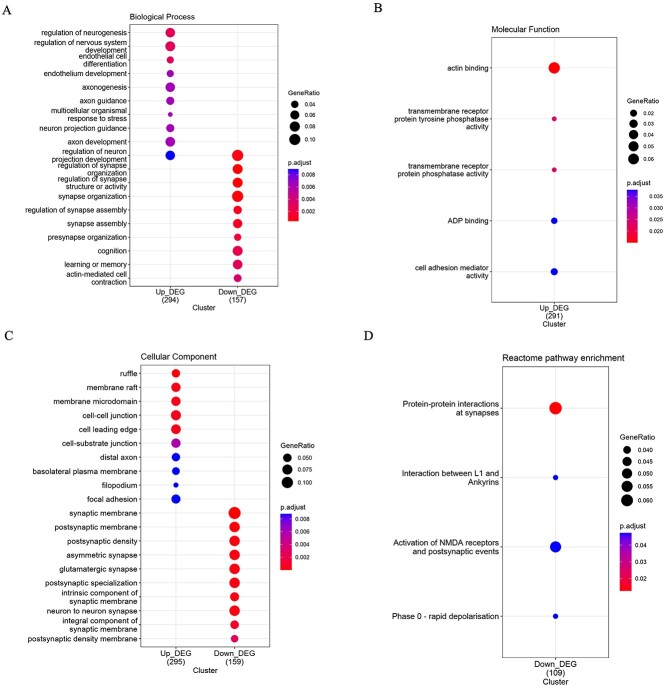
Gene enrichment analyses based on (A) Biological process (BP), (B) Molecular function (MF), (C) Cellular components (CC), and (D) Reactome pathway enrichments. Significant enrichment terms were selected based on the adjusted *P*-value <0.01 and p.adjust.methods = ‘BH’. BH: Benjamini- Hochberg.

The effective gene enrichment terms were selected based on an adjusted *P*-value <0.01, with the top 20 enrichment terms presented in Figure 5. Gene enrichment analysis for biological process (BP) revealed that the up-regulated DEGs primarily regulated Neurogenesis. In contrast, the down-regulated DEGs were associated with the regulation of synapses (Figure 5A). For molecular function (MF), a few enrichment terms, including actin-binding, transmembrane receptor protein tyrosine phosphatase activity, and transmembrane receptor protein phosphate activity, were significantly enriched only for the down-regulated DEGs (Figure 5B). Regarding cellular components (CC), the up-regulated DEGs were associated with processes such as membrane raft and membrane microdomain, cell–cell junction, cell leading edge, and cell-substrate junction. In contrast, the down-regulated DEGs were enriched in synapse-related terms such as postsynaptic membrane, postsynaptic density, asymmetric synapse, and glutamatergic synapse (Figure 5C). Furthermore, PPI at synapses was identified as the most significant Reactome pathway associated with the down-regulated DEGs. In contrast, no pathways were found to be significantly associated with the up-regulated DEGs in this study (Figure 5D).

The relationship between down-regulated DEGs and synapse regulation at D54 in human embryonic stem cell development is significant for understanding the molecular mechanisms underlying neuronal differentiation and synaptic maturation. Studies have shown that the down-regulation of specific genes can impact synapse formation and function during this critical developmental stage. At D54, the down-regulation of certain DEGs can influence synaptic development by modulating critical pathways involved in synapse regulation. For example, the decreased expression of genes associated with synaptic plasticity, neurotransmitter release, or dendritic development can affect the establishment and maturation of synapses in developing neurons [39]. This intricate interplay between gene expression changes and synaptic regulation is crucial for shaping the connectivity and functionality of neuronal networks.

Moreover, the dysregulation of gene expression patterns at D54 can disrupt the intricate balance required for proper synapse formation and function, potentially leading to aberrant synaptic connectivity and neuronal communication. Understanding how specific DEGs influence synapse regulation at this stage provides valuable insights into the molecular underpinnings of neuronal maturation and the establishment of functional neural circuits in human embryonic development [39]. A recent study identified groups of DEGs with distinct patterns in up-regulated and down-regulated genes, where up-regulated DEGs were enriched in cell cycle pathways. In contrast, down-regulated DEGs were associated with neuronal pathways related to synaptic transmission [40].

### DEGs involved in neurodevelopmental disorders

**Figure 6:**
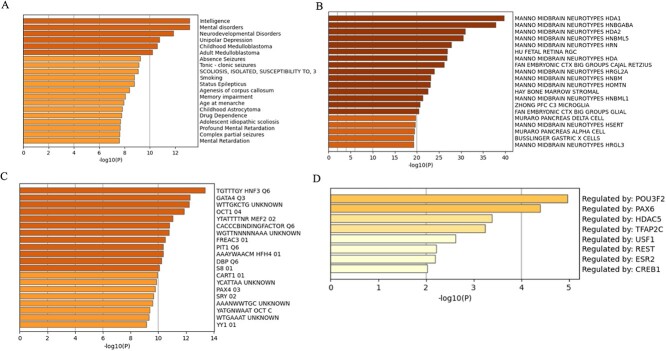
Summary of enrichment analysis using the DEGs. (A) DisGeNET, (B) Cell Type Signatures, (C) Transcription Factor Targets, and (D) TRRUST.

We investigated the enrichment analysis in DisGeNET [41], cell type signatures [42], transcription factor targets and TRRUST [43]. Results showed that intelligence, mental disorders and neurodevelopmental disorders are the most significantly enriched terms in DisGeNET (Figure 6A). Cell Type Signatures analysis showed that the top five enrichment terms are related to NEUROTYPE (Figure 6B).

In the context of HNF3 Q6, genes containing the 3´-UTR motif TGTTTGY exhibit the most notable enrichment among TF targets (Figure 6C). Research showed that the *FOXA1*, *Forkhead Box A1*, is a TF involved in embryonic development and protein-coding genes related to several pathways, including embryonic and lineage-specific markers (www.genecards.org). DEGs are regulated by *POU3F2*, *PAX6*, *HDAC5*, *TFAP2C*, *USF1*, *REST*, *ESR2* and *CREB1* in TRRUST (Figure 6D). The study showed that enrichment terms in TRRUST were associated with brain development, disease, or progression of tumors (Table S3, see Supplementary Data are available online at http://bib.oxfordjournals.org/).

We also investigated the DEG expression in different tissues using the GTEx database. Results showed that most of the up-regulated DEGs are highly expressed in multiple brain tissues, including the hypothalamus, amygdala, putamen, hippocampus, cortex, cerebellum and cerebellar hemisphere (Figure S3, see Supplementary Data are available online at http://bib.oxfordjournals.org/). Although the down-regulated DEGs were also expressed in different brain tissues mentioned above, the expression levels were relatively low, except in the cerebellum and cerebellar hemisphere, compared with up-regulated DEGs (Figure S3, see Supplementary Data are available online at http://bib.oxfordjournals.org/).

### Functional network discovery through PPI analysis

**Figure 7:**
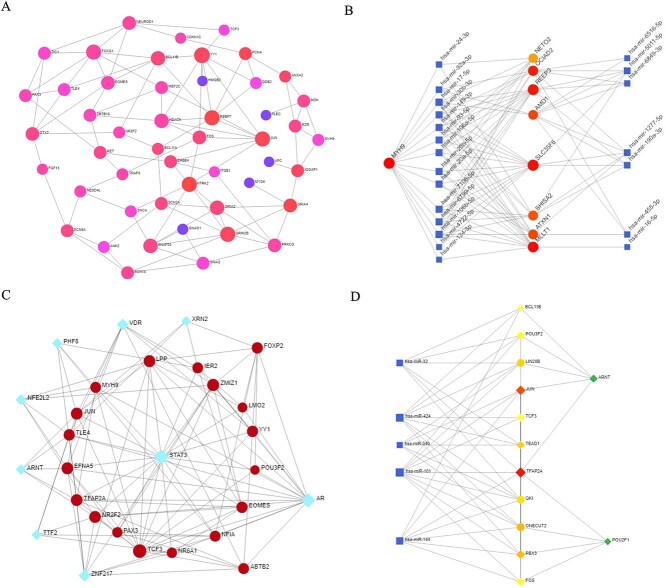
Different network plots. (A) PPI network plot, (B) DEGs-miRNAs network plot, (C) TF-DEGs network plot, and (D) TF-miRNA network plot. These GRN analyses identified interactions between important biomarkers and their association with miRNAs and TFs.

The PPI network was generated using STRING's web-based visualization resource [29]. A cutoff value of 900 was employed, and this network was constructed based on experimental evidence (Figure 7A). Circular nodes received color assignments based on their degree within the network. For example, YY1, NTRK2, RBBP7, JUN, GRIN2B and FOXG1 exhibited the highest significance in the PPI analysis (Figure 7A).

### Revealing regulatory cross-talks among DEGs, miRNA, and TF interactions using gene regulatory networks

In our gene regulatory network (GRN) analysis, we employed the associated DEGs of the transcriptional factors and microRNAs responsible for regulating these DEGs at the post-transcriptional level. We identified DEG–miRNA interactions using miRTarBase bases, and the network is presented in Figure 7B. The circles and squares represent the DEGs and miRNA, respectively, in the figure (Figure 7B). Colors were assigned to circular nodes based on their degree within the network. The node degree represents the number of edges connected to it. Nodes with higher degrees are regarded as central hubs in the network, and we have highlighted their sizes for emphasis. For instance, nodes with red coloration, including *MYH9*, *MLLT1*, *SLC35F6*, *AMD1*, *REEP3* and *OCIAD2*, exhibit heightened significance (Figure 7B).

We also depicted the interaction network involving TF–DEGs. This network is visually presented in Figure 7C, where circular nodes represent DEGs and diamond-shaped nodes signify TFs. Node size within the network corresponds to the node's degree, signifying its connectivity. Notably, genes like *TCF3* and *ZMIZ1*, which exhibit a higher degree, are more prominently expressed among the DEGs (Figure 7C). Additionally, our analysis highlights the prominence of certain transcription factors, such as *STAT3* and *AR*, as evidenced in the same figure. The resulting TF-miRNA interaction network is visually depicted in Figure 7D, with square nodes representing miRNAs and diamond-shaped nodes denoting TFs. The size of each node in the network reflects its degree, indicating the extent of its connectivity. We found that only two TFs, *ARNT* and *POU2F1*, stand out as more highly connected nodes within the network, highlighting their significance in the TF-miRNA regulatory landscape (Figure 7D).

### Transcription factor enrichment analysis

**Figure 8:**
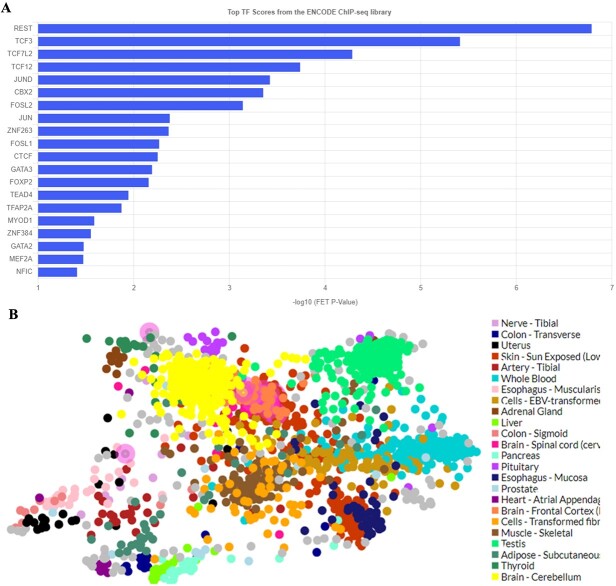
Transcription factor enrichment analysis results. (A) Bar chart of the top 20 TFs scores from the ENCODE ChIP-seq library, and (B) Network using the GTEx TF data and the colored by tissues.

In performing TF enrichment analysis, we have used ChEA3, which is used to forecast TFs linked to user-provided gene sets. To establish potential associations between the input gene set and TFs, the Fisher's Exact Test was employed. We identified the top 20 TFs in our study (Figure 8A). We have also investigated the TF co-expression networks to understand better the significance of the top-ranked TFs in the broader human transcriptional regulatory network. The color choices for network nodes offer extra insights into the tissues or types of tumors where the TFs might be most active. Results showed that most of the TFs were active in the cerebellum region of the brain (Figure 8B).

### Comparison of DEG detection methods

In our comparative analysis, we evaluated our proposed method alongside the recently updated DEsingle [44]. DEsingle, an R package designed for analyzing scRNA-seq data, introduces a novel approach using the Zero-Inflated Negative Binomial model [44]. This model effectively distinguishes between ‘real’ zeros and ‘dropout’ zeros in gene expression profiles. Notably, DEsingle outperformed existing methods in performance validation, demonstrating its accuracy and utility [44]. Additionally, we explored another method called SigEMD [45]. SigEMD is a novel approach for analyzing differential gene expression in scRNA-seq data [45]. It addresses challenges related to multimodality, zero counts, and sparsity by combining data imputation, logistic regression, and the nonparametric Earth Mover’s Distance [45]. SigEMD’s performance surpasses existing methods in terms of precision, sensitivity, and specificity, making it a robust tool for scRNA-seq analysis [45].

**Figure 9:**
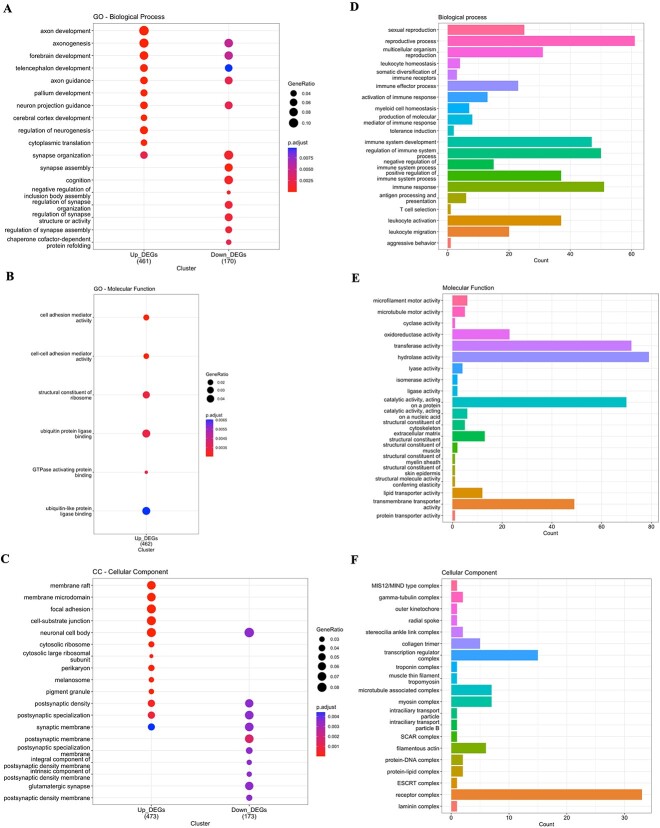
Gene enrichment and Gene Ontology (GO) analysis results by DEsingle and SigEMD. (A-C) Biological Process (BP), Molecular Function (MF), and Cellular Components (CC) results of the DEGs identified by the DEsingle method, respectively. (D-F) BP, MF, and CC results of the DEGs identified by the SigEMD method, respectively. This figure provides insights into the functional annotations and cellular localization of the identified DEGs using both methods.

We have identified a total of 822 DEGs, where 576 were up-regulated and 246 were down-regulated DEGs, determined by the DEsingle method (Table S4, see Supplementary Data are available online at http://bib.oxfordjournals.org/). Enrichment analysis of BP results showed that up-regulated DEGs were enriched in axon development, axonogenesis, forebrain development, neuron projection guidance, regulation of neurogenesis and other processes (Figure 9A). On the other hand, down-regulated DEGs are mostly enriched in synapse organization, synapse assembly, regulation of synapse organization, regulation of synapse structure or activity and regulation of synapse assembly (Figure 9A). Enrichment analysis of MF results showed that only down-regulated DEGs were enriched in cell adhesion mediator activity, cell–cell adhesion mediator activity, structure constituent of the ribosome and ubiquitin protein ligase binding (Figure 9B). Enrichment analysis of CC results showed that up-regulated DEGs were enriched in membrane raft, membrane microdomain, focal adhesion and neuronal cell body (Figure 9C). On the other hand, down-regulated DEGs are mostly enriched in the synaptic membrane and postsynaptic membrane (Figure 9C).

However, the SigEMD method identified 1196 DEGs in total—with distinct GO terms compared with our proposed method (Figure 9D-F and Table S5, see Supplementary Data are available online at http://bib.oxfordjournals.org/). Notably, SigEMD does not categorize DEGs into up- and down-regulated groups. We explored BP, MF and CC annotations using all DEGs identified by SigEMD for further insights into the molecular mechanisms underlying changes in gene expression. Functional profile results in the BP showed that DEGs were enriched in the reproductive process, regulation of the immune system and immune response (Figure 9D). DEGs in the MF were enriched in the transferase activity and transmembrane transporter activity (Figure 9E). DEGs in the CC were enriched in the transcription regulator activity and receptor complex (Figure 9F). The DEGs identified by DEsingle exhibited similar biological terms, reinforcing the consistency and reliability of our proposed approach. The DEGs identified by the SigEMD method with distinct GO terms compared with our proposed method. The analysis provides valuable information on the functional roles of genes and the biological pathways of their products. These findings contribute to our understanding of gene expression dynamics in single-cell RNA-seq data.

## DISCUSSION

Studying neuron development from the early stage of human embryonic brain expansion is crucial to understanding human brain development. However, the influences of the genetic variants during the early stage of human brain development using the scRNA-seq data from the hESCs have not been exhaustively investigated. In this study, we have utilized 762 hESCs cells to identify the important DEGs between two time points (D26 and D54) to understand the brain complexity at the single-cell level. We employed a multi-pronged approach to identify DEGs within our scRNA-seq dataset. Leveraging the power of three distinct statistical methods, MAST, limma, and DESeq2, we accurately scrutinized the gene expression changes across the dataset. To ensure the robustness and accuracy of the DEGs for further investigation, we focused on the common DEGs identified by all three methods. Finally, we have used 539 DEGs for the downstream analysis in our study.

We have found that some up-regulated DEG is involved in all of the BP, MF and CC processes. For example, *NRP1*, *LHX2* and *KIAA0319* are up-regulated DEGs involved in BP processes. Research showed that gene *NRP1* (neuropilin 1), expressed in neurons, was also found to be expressed in arteries, immune cells and numerous different cell types and involved in a range of anatomically and functionally various extracellular ligands to regulate organ expansion and function [46]. A study showed that the function of *LHX2*, LIM-homeobox 2, in regulatory neuronal against glial fate choice is complex and is an important factor for the expansion of retinal Muller glia differentiation [47]. A recent investigation showed that *LHX2* plays a significant role in cell differentiation, cell signaling, tissue-type differentiation, and body development [48]. Another up-regulated DEG, *KIAA0319*, determines the movement of the cell, cilia length and mechanical cell-surface contact [49]. Studies showed that micro-RNAs play crucial functions in tumor invasion, propagation, and the expression of *ITGB1,* reducing tumor cells' ability to attack and metastasize [50].

In contrast, most of the down-regulated DEGs were associated with brain development, and some of them played roles in all of the BP and CC processes. For example, down-regulated DEG *ADGRB3* plays a central role in controlling various mechanisms of the central nervous system, including synapse creation and function, axon regulation and myelination [51]. The structure of a neuron helps its functionality inside neural circuits, and the neuron starts to move in the direction of the cortical plate during embryonic development. The study showed diverse progress of cortical neurons and dendrites controlled by the *EphA7* [52]. A neuron is morphologically complicated and depends deeply on its exceptional cytoarchitectural networks, such as axons and dendrites, to conduct its functions. Neurons rely on various transportations and expressions of proteins at distinct subcellular chambers to retain their morphological arrangement [53]. Many neurophysiological procedures, such as neuroprotection, expansion of neurons and glial cells, and modulation of synaptic relations, are influenced by the brain-derived neurotrophic factor (*BDNF*) [54]. Its spatiotemporal expression controls the influences of BDNF and its connection with neurotrophic receptor tyrosine kinase 2 (*NTRK2*) and neurodegenerative disorder caused by the mutations of BDNF in humans [55, 56]. *NTRK3* is crucial to the development of the nervous systems, cancer and tumor formation and advancement stimulated by the modifications of NTRK3 [57].

*ADCY1*, *adenylate cyclase 1,* is a neuron-specific protein catalyzing cAMP production and is preferably enriched at the postsynaptic viscosity [58]. The calcium and neuronal provocation could govern the activity of the *ADCY1*. Therefore, the regulation of neuronal signal transduction and synaptic moldability could be directed by the function of *ADCY1* [59]. The accurate governance of the nervous system process under the dynamism of synapses is particularly crucial. The cell adhesion molecules could influence the synapse congregation in the brain. A study showed that *ErbB4* stimulates repressive synapse development by cell adhesions and is enhanced in interneurons [60]. Synapse creation and neuronal expansion could be controlled by the involvement of the LRFN family and their extracellular domain [61, 62]. *LRFN5*, leucine-rich repeat and fibronectin type III domain-containing 5 may affect both inhibitory and excitatory presynaptic distinction in proximate neuronal cells. Also, *LRFN5* might have a significant role in brain growth and procedure [63]. Genes with uniform expression levels over the periods could act as targets in an overall mode, while some neuron development-associated DEGs expressed in a specific time point could serve as specific regulation of neuronal development pathways in particular time points in the process of human brain development. These results indicate that these DEGs are necessary for neuronal development throughout time.

We have performed a PPI network analysis. The highest significant DEGs are involved in the diverse brain development pathways. For example, *YY1* is a transcription factor that regulates cell proliferation, differentiation, and apoptosis [64]. YY1 regulates embryonic development, hematopoiesis and cancer development [64]. A recent study suggests that YY1 can be used to modulate stem cells as a potential treatment for severe mental disorders and cognitive impairments [65]. *NTRK2*, which encodes the neurotrophic tyrosine receptor kinase 2, belongs to the neurotrophic tyrosine receptor kinase family. The NTRK2 protein acts as a membrane-bound receptor, and upon binding with neurotrophins, it phosphorylates itself and activates members of the MAPK pathway. Activation of this kinase initiates cell differentiation. NTRK2 regulates neurogenesis, synaptogenesis, and neuronal plasticity (https://www.genecards.org/).

A study suggests that genetic variability of NTRK2 is related to emotional arousal and the integrity of white matter in the brains of healthy young individuals [66]. *RBBP7* is a nuclear protein with ubiquitous expression, forming a part of a conserved subfamily of WD-repeat proteins. It regulates chromatin structure and gene expression and is essential for DNA replication, repair and cell cycle progression [67]. It has been shown to regulate cell proliferation and histone H3.3 placement during early mouse embryo development [67]. *JUN* is a proto-oncogene encoding a transcription factor in the AP-1 family, regulating cell proliferation, differentiation, apoptosis, immune response, inflammation, and cancer development (https://www.genecards.org/). *GRIN2B* encodes the GluN2B subunit of NMDA receptors, and the study identified that disruption of GRIN2B impairs differentiation in human neurons [68]. *FOXG1*, encoding the forkhead box G1 protein, is required to regulate neural stem cell proliferation, differentiation, cerebral cortex development and olfactory bulb development [69]. This transcription repression factor assumes a vital role in shaping the distinct regions of the developing brain and is instrumental in forming the telencephalon (https://www.uniprot.org/uniprot/P55316#function). These findings provide valuable insights into the multifaceted roles of these genes, with potential implications for understanding and treating various health conditions.

We also found links between different diseases by examining how they relate through protein interactions, gene-miRNA interactions, TF-miRNA interactions and TF-gene interactions. For example, the gene *MYH9*, which shows a strong association with miRNAs, encodes a protein known as myosin-9, a subunit of the myosin IIA protein [70]. A recent study found that MYH9 is pivotal for the survival and upkeep of hematopoietic stem/progenitor cells (HSPCs). Its deletion results in diminished HSPC repopulation capacity and elevated apoptosis [71]. The hsa-mir-17-5p is a microRNA that regulates cell proliferation, differentiation, and apoptosis [72]. A recent study identified that miR-17-5p contained exosomal derived from hESCs, which prevents pulmonary fibrosis through interaction with thrombospondin-2 [73]. MicroRNA hsa-mir-16-5p plays a critical role in regulating gene translation by silencing or degrading target mRNAs (https://www.biovendor.com/mir-16-5p). Research showed that hsa-mir-16-5p suppresses myoblast proliferation, enhances myoblast apoptosis, represses myoblast differentiation and facilitates changes in apoptosis-related gene expression [74]. Studies showed that miR-30b-3p has been shown to regulate glucose and lipid metabolism in adipocytes and hepatocytes, contributing to the regulation of insulin secretion and sensitivity [75]. Hsa-mir-149-3p controls the transition from fat cell development to bone cell development in mesenchymal stem cells by regulating FTO [76].

TFs are key regulators of cellular processes, each with distinct roles. In our investigation of hESCs, we focused on the RE1-silencing transcription factor (REST), which controls gene expression by binding to RE1 sites in DNA [77]. REST contributes to shaping neuronal development and function and it has also been implicated in the pathogenesis of neurological disorders such as epilepsy, Alzheimer's disease and Huntington's disease [77]. JUND, part of the AP-1 transcription factor family, oversees vital cellular functions like growth and differentiation and plays a role in regulating immune responses, inflammation, and cancer development [78]. FOXP2, a transcription factor, controls speech, language and nervous system development [79]. It also manages neurogenesis, synaptogenesis and neuronal plasticity [79]. MEF2A acts as a critical transcription factor and plays an integral part in regulating muscle differentiation, hypertrophy and the process of regeneration [80]. CTCF stands out for its role in regulating chromatin structure and gene expression, encompassing functions like genomic imprinting, X-chromosome inactivation and long-distance chromatin interactions [81]. CBX2, on the other hand, directs chromatin architecture, wielding its influence over embryonic stem cell differentiation, X-chromosome inactivation and heterochromatin formation [82]. TCF12 governs essential cellular behaviors, impacting neurogenesis, craniofacial development and cancer development.

Meanwhile, TFAP2A plays a crucial role in activating placental genes and repressing the pluripotency gene OCT4, thus orchestrating the transition from trophoblast specification to departure from pluripotency [83]. Studies have shown that FOSL1 and FOSL2 are key TFs pivotal in orchestrating pluripotency and differentiation of human T helper 17 (Th17) cells [84]. GATA2 and GATA3 are identified as crucial TFs in the establishment and sustenance of hematopoietic systems [85]. GATA2 plays a critical role in promoting the proliferation and viability of lineage-specific transcription as a dynamic partner of GATA1. In contrast, GATA3 is essential for the development of T lymphoid cells and contributes to immune regulation [85]. Research showed that TCF7L2 serves as a master regulator in the vertebrate brain, governing stage-specific genetic programs and maintaining a regional transcriptional network throughout embryonic development. It also plays a role in the postnatal phase [86]. TCF3 is a vital element within the regulatory network of embryonic stem cells. Essentially, it facilitates the transmission of development signals from the Wnt pathway to the central regulatory network of embryonic stem cells, thereby impacting the equilibrium between pluripotency and differentiation [87].

The novelty of this study is that scRNA-seq data is used to pinpoint DEGs at two critical time points, D26 and D54, in hESCs, providing a detailed understanding of genetic dynamics during early brain development. Another novelty of this study is that it performs gene enrichment analysis and reveals functional enrichment in pathways such as neurogenesis and synapse regulation, unraveling the intricate processes that govern early brain development. Also, this study explores the enrichment analysis of the DEGs in terms of intelligence, mental disorders, and neurodevelopmental disorders, offering valuable insights into the intersection of early brain development and cognitive outcomes. Furthermore, this study explores interactions among DEGs, miRNAs and TFs, unveiling their roles in shaping early neuronal development. Moreover, this study identifies the top 20 TFs and their co-expression networks in the cerebellum region of the brain.

In conclusion, the DEG biomarkers unveiled by our investigation have been found to play a pivotal role in the arrangement of gene expression during neural differentiation. Their functional purview extends to the intricate governance of neural cell development, encompassing a spectrum of regulatory functions. In this symphony of genetic activity, the revelations from our single-cell gene expression analysis furnish valuable insights into the molecular mechanisms underpinning neural differentiation, elevating our understanding of this complex cellular phenomenon. In the future, we will expand the scRNA-seq dataset to include more time points and cell types during early brain development, as well as more biological replicates, to increase the statistical power and robustness of the analysis. In addition, the DEGs and their regulatory networks need to be validated using experimental methods such as qRT-PCR, ChIP-seq and CRISPR-Cas9 to confirm their expression patterns and functional roles in neural differentiation and brain development. Furthermore, more study is required in order to investigate the potential applications of the DEGs and their regulators for developing novel diagnostic biomarkers, therapeutic targets and stem cell-based interventions for various neurological disorders and cognitive impairments.

**Key Points**

- This study utilizes bioinformatics tools to pinpoint DEGs in human embryonic stem cells (hESCs) at key developmental time points, D26 and D54, providing a nuanced understanding of genetic dynamics during early brain development.
- Through subsequent analyses, the research reveals functional enrichment in critical pathways such as neurogenesis and synapse regulation, unraveling the intricate processes that govern early brain development.
- Investigating potential links of the identified DEGs to intelligence, mental disorders, and neurodevelopmental disorders, offering valuable insights into the intersection of early brain development and cognitive outcomes.
- Delving into the regulatory landscape, the research explores interactions among DEGs, miRNAs and TFs, unveiling their roles in shaping early neuronal development.
- The findings collectively showcase the power of scRNA-seq in unraveling the complexities of early human brain development.

## Supplementary Material

Supplementary_Figures_bbae230

Supplementary_data_bbae230

## Data Availability

All data analyzed in this study were published previously [8] and available in NCBI Gene Expression Omnibus (accession no: GSE86982) at https://www.ncbi.nlm.nih.gov/geo/query/acc.cgi?acc=GSE86982.

## Author Biographies

**Md. Alamin** is a Senior Research Scholar at the Shenzhen Key Laboratory of Gene Regulation and Systems Biology, School of Life Sciences, Southern University of Science and Technology, Shenzhen, 518055, China. He completed his Ph.D. from the Zhejiang University in 2018. His research interests are in bioinformatics, single-cell genomics, and machine learning.

**Most. Humaira Sultana** received her Ph.D. from the Zhejiang University, Hangzhou 310058, China. Her research interests are in bioinformatics, computational biology and machine learning.

**Isaac Adeyemi Babarinde** is a Senior Research Scholar at the Shenzhen Key Laboratory of Gene Regulation and Systems Biology, School of Life Sciences, Southern University of Science and Technology, Shenzhen, 518055, China. His research interests are in bioinformatics.

**AKM Azad** is an Assistant Professor at the Department of Mathematics and Statistics, College of Science, Imam Mohammad Ibn Saud Islamic University (IMSIU), Riyadh 11432, Saudi Arabia. He received a Ph.D. degree from Monash University, Australia in computational systems biology and biostatistics in 2017. His research interests include AI/ML/DL, Bayesian statistics, MCMC, Bayesian networks, bioinformatics and computational biology.

**Mohammad Ali Moni** is an AI & Digital Health Technology Lead, Artificial Intelligence and Cyber Futures Institute, Charles Stuart University, Bathurst, NSW 2795, Australia. His research interests encompass artificial intelligence, machine learning, data science and clinical bioinformatics.

**Haiming Xu** is a professor at the Institute of Bioinformatics, College of Agriculture and Biotechnology, Zhejiang University, Hangzhou 310058, China. His research interest is in Bioinformatics and Statistical genetics.
