# Supplementary material for: Single-cell RNA-seq data analysis reveals functionally relevant biomarkers of early brain development and their regulatory footprints in human embryonic stem cells (hESCs)
Source: Brief Bioinform. 2024 May 12;25(3):bbae230. doi: 10.1093/bib/bbae230 (PMC11089419; doi:10.1093/bib/bbae230)
Supplement: Supplementary_Figures_bbae230 [file supplementary_figures_bbae230.docx]

**Supplementary Figures**

**
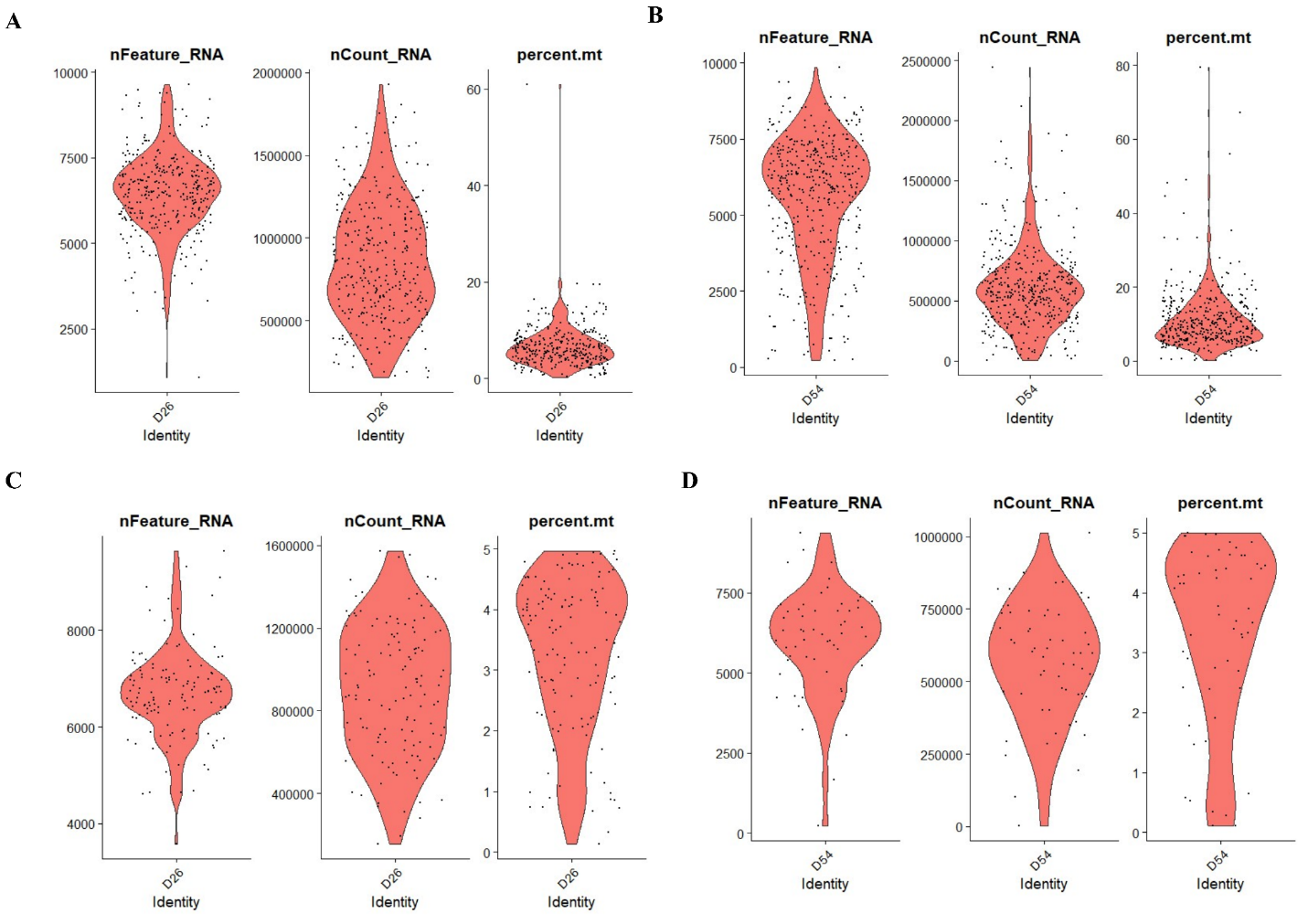
**

**Figure S1.** Violin plots show quality control (QC) metrics before (A, B) and after (C, D) QC for the data at D26 and D54, respectively. We filtered out cells with unique feature counts over 10,000 or less than 200 and cells with mitochondrial counts greater than 5%.


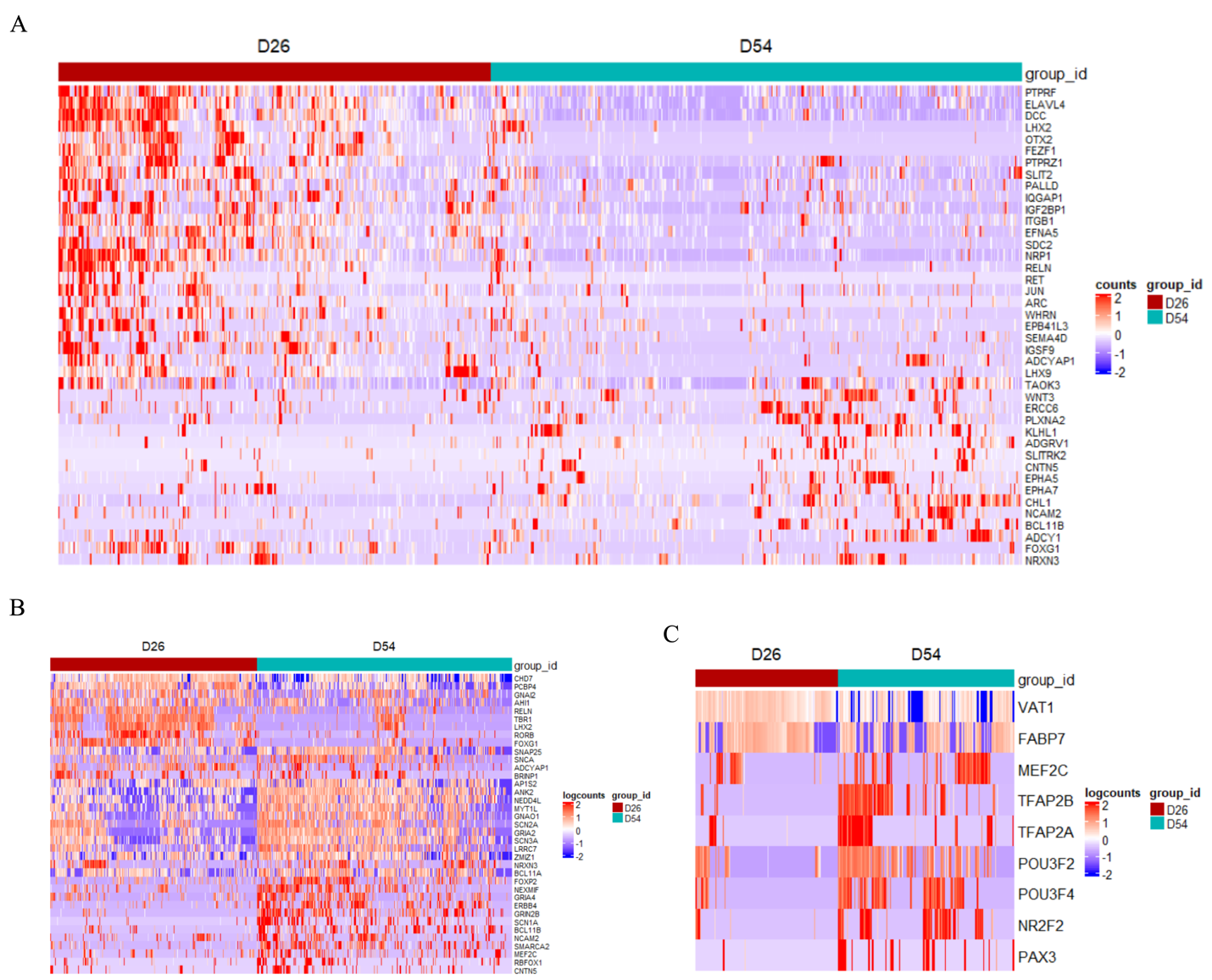


**Figure S2.** Heat map using (A) neuron projection development, (B) neuron development disorder, and (C) progenitor and neuron development-related DEGs.

**
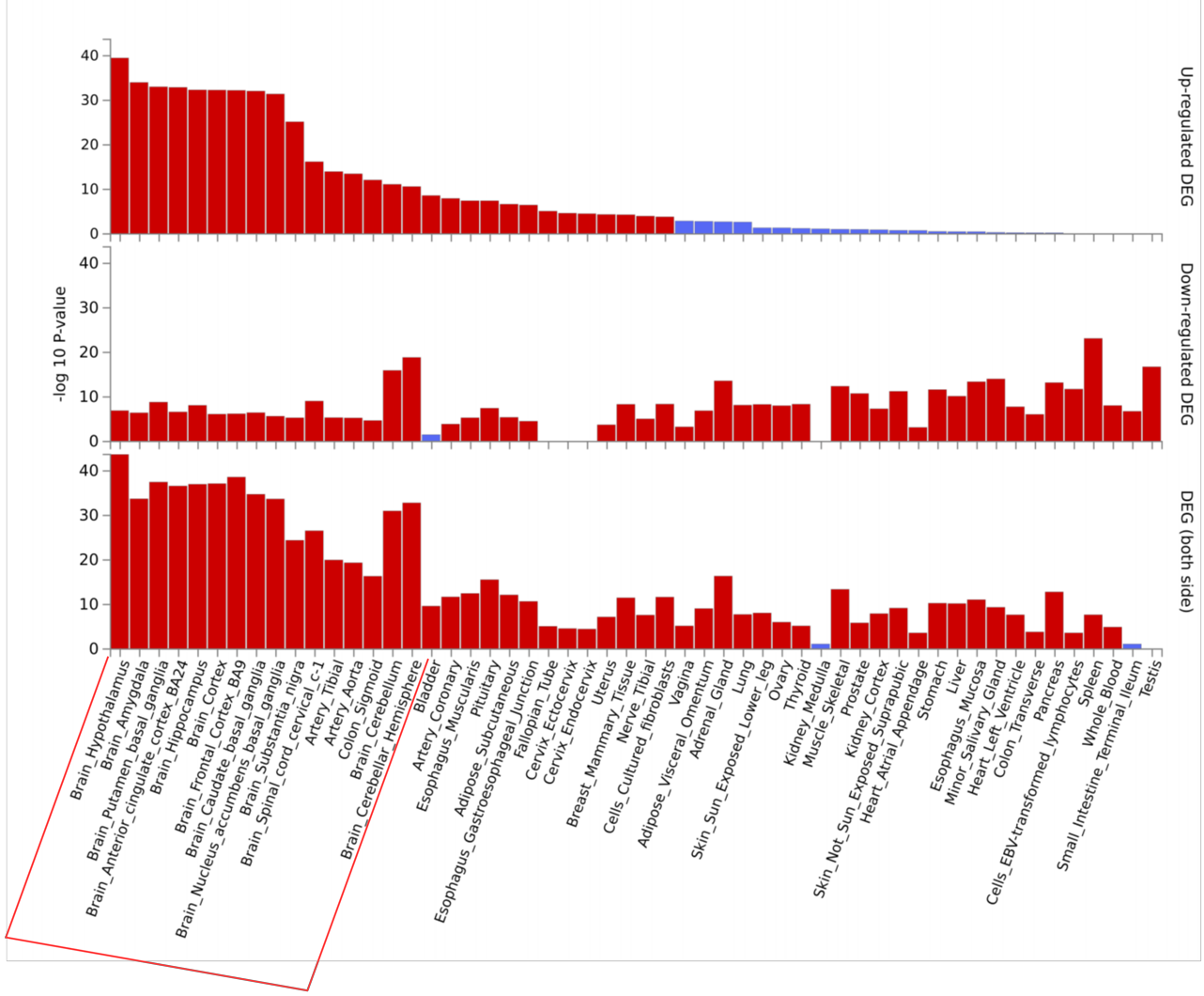
**

**Figure S3.** Expression at different tissues (GTEx v8 54 tissue types) of the differentially expressed genes (DEGs). Significantly enriched DEG sets (P_bon_< 0.05) are highlighted in red.
